# Critical Shear Stress is Associated with Diabetic Kidney Disease in Patients with Type 2 Diabetes

**DOI:** 10.1038/s41598-018-19274-5

**Published:** 2018-01-17

**Authors:** Seung Min Chung, Jung Hyun Oh, Jun Sung Moon, Yu Kyung Kim, Ji Sung Yoon, Kyu Chang Won, Hyoung Woo Lee

**Affiliations:** 1 Division of Endocrinology and Metabolism, Department of Internal Medicine, Yeungnam College of Medicine, Daegu, Republic of Korea; 2 Kwon and Oh Internal Medicine, Sangju, Gyeongbuk, Republic of Korea; 3 Department of Clinical Pathology, School of Medicine, Kyungpook National University, Daegu, Republic of Korea

## Abstract

Critical shear stress (CSS, mPa) is an index of red blood cell (RBC) aggregability, defined as the minimal shear stress required to disperse RBC aggregates. This study aimed to investigate the association between CSS and the risk of diabetic kidney disease (DKD). A total of 421 (mean age, 58.1 ± 11.5 years; male, 250) individuals with T2DM were enrolled and divided into three groups according to CSS level. CSS was measured using a transient microfluidic technique. DKD was defined as a glomerular filtration rate (GFR) <60 ml/min/1.73 m^2^ or a urine albumin-to-creatinine ratio (uACR) ≥30 mg/g. CSS was significantly higher in patients with DKD than in those without (317.43 ± 125.11 vs 385.22 ± 182.89, p < 0.001). Compared to the lowest CSS tertile, the highest CSS tertile was independently associated with the risk of DKD after adjusting for age, sex, duration of diabetes, presence of hypertension and haemoglobin. The cut-off value of CSS for DKD was approximately 310 mPa. These results suggest that haemorheologic changes may contribute to DKD, and further prospective studies are warranted to determine the role of CSS as a DKD screening tool.

## Introduction

The prevalence of type 2 diabetes mellitus (T2DM) is increasing worldwide. In 2015, 8.8% (415 million) of the global population aged between 20 and 79 years was estimated to have diabetes, and the disease is expected to increase 1.5 times to 2040^1^. In Korea, the prevalence of diabetes in those aged 30 years and over is estimated as 13.7% (4.8 million) and in those over 65 years of age as 30%^2^. Diabetic micro- and macro-vascular complications are a major cause of mortality in T2DM patients. Micro-vascular complications consist of diabetic retinopathy (DR), kidney disease (DKD), and peripheral neuropathy (DPN). Micro-vascular complications, especially DKD, can be a risk factor for macro-vascular complications such as atherosclerosis, myocardial infarction, stroke, and heart failure^3^. Therefore, the effort to screen for micro-vascular complications, including DKD, is essential to prevent the progression to macro-vascular complications and a deterioration in the quality of life^4^.

T2DM, hypertension, obesity and dyslipidaemia, also commonly referred to as metabolic syndrome, are status indicators of oxidative stress and chronic inflammation that alter haemorheology, decrease RBC deformability, alter RBC morphology, increase RBC aggregation, and increase plasma viscosity^5^. In T2DM, advanced glycation end products (AGE) play a critical role in haemorheologic change. RBCs produce AGEs, and many adhesion molecules are expressed in the vessels’ endothelial cells, and their increased cohesion induces oxidant stress^6^. The correlation between haemorheological alterations and either diabetic micro- or macro-vascular complications has been recognised, and related studies have been reported. RBC deformability showed a significant decrement in DR^7^ or DKD^8,9^ while RBC aggregability and plasma viscosity showed a significant increment in acute coronary syndrome^10^, diabetic peripheral arterial occlusive disease^11^, or diabetic foot disease^12^.

RBC aggregability is shear-dependent, and increased aggregability affects the flow properties of RBCs in the microcirculation^13,14^. Critical shear stress (CSS, mPa), the minimal shear stress required to disperse RBC aggregates, has been recently suggested as an index of RBC aggregability^15^. Although reversible RBC aggregates can be easily observed in venules in a status of either stasis or high-to-low shear flow conditions, RBC aggregation in arteries is considered pathological, which may result in a worsening change in the clinical course^15,16^.

The association between diabetic micro-vascular complications and RBC deformability has been revealed in several previous studies; however, the association with CSS is little known. For this reason, this study was designed to investigate the association between CSS and the risk of diabetic micro-vascular complications.

## Participants and Methods

### Study population

This cross-sectional and retrospective study enrolled 456 T2DM inpatients and outpatients who visited Yeungnam University Hospital (Daegu, Korea) between September 2014 and May 2017. Data and samples were collected at baseline visit. To exclude factors that can confound kidney function markers without kidney damage^17^, such as aging, liver disease and infection, or those that can affect statistical variance in analysis, the criteria for exclusion were as follows: over 85 years of age (n = 2); acute inflammation or infection (white blood cell ≥20,000/µL or high-sensitivity c-reactive protein (hs-CRP) ≥10 mg/dL, n = 8); anaemia (haemoglobin [Hb] ≤8 g/dL, n = 1); impaired liver function (aspartate aminotransferase ≥100 or alanine aminotransferase ≥100, n = 16); hypertriglyceridemia (triglyceride [TG] ≥1000 mg/dL, n = 2); and patients without blood urea nitrogen (BUN) or creatinine (n = 6) results. Finally, 421 participants were enrolled in this study.

All patients gave informed consent, and approval was obtained from the local ethics committee. The Institutional Review Board of Yeungnam University Hospital approved the study protocol. All experiments were performed in accordance with relevant guidelines and regulations.

### Diabetic complications

All participants were examined for the presence of diabetic micro-vascular complications^18^. DR was defined as the presence of macular oedema, dilated veins, microaneurysm, haemorrhage, or vessel proliferation identified with retinal photography. DR was classified as normal, non-proliferative diabetic retinopathy (NPDR); proliferative diabetic retinopathy (PDR) was characterised by the growth of new blood vessels on the retina and on the posterior surface of the vitreous^19^. DKD was defined as the presence of albuminuria and/or a reduced estimated glomerular filtration rate (eGFR) in the absence of signs or symptoms of other primary causes of kidney damage, a urinary albumin-creatinine ratio (uACR) ≥30 mg/g, and/or an eGFR <60 mL/min/1.73 m^2^. In detail, a uACR 30–300 mg/g was defined as moderately increased albuminuria and a uACR >300 mg/g as severely increased albuminuria^17^. The eGFR was calculated using the MDRD formula: 186 × (creatinine)^−1.154^ × (age)^−0.203^ × (0.742 if female) × (1.210 if black). DPN was diagnosed using a 10 g monofilament and assessing clinical scores on a questionnaire (Michigan Neuropathy Screening Instrument)^20^.

### Measurements

Body mass index (BMI) was calculated as body weight in kilograms divided by height in meters squared (kg/m^2^). Diabetes duration was measured in years. Blood pressure was measured twice in the sitting position, and a mean value was calculated.

All laboratory parameters were determined in the central laboratory of the Yeungnam University Hospital. Venous sampling was taken from the antecubital vein after an overnight fast. The levels of serum glucose, glycated haemoglobin (HbA1c), Hb, total cholesterol (T-cho), high-density lipoprotein cholesterol (HDL-cho), low-density lipoprotein cholesterol (LDL-cho), TG, BUN, creatinine, erythrocyte sedimentation rate (ESR), hs-CRP, fibrinogen were measured. A urine test was performed to measure uACR. uACR test was repeated three times within 12 months if elevated over 30 mg/g, and increased urinary albumin excretion was confirmed when at least two results were elevated over a 3-to 6-month period^18^.

### Hemorheologic parameters

CSS was measured with a transient microfluidic haemorheometer (Rheoscan-D, Sewon Meditech Inc., Seoul, Korea), using native whole blood without adjusting for haematocrit. Whole blood sample (500 µl) was stored in a reservoir chamber and sheared in the microchannel under continuously decreasing pressure differentials. As the pressure differential decreases, the RBC aggregates tend to disperse at high shear flows, and the corresponding backscattered light (BSL) intensity increases. However, as the pressure differential decreases further, the dispersed RBCs re-aggregate and the BSL intensity decreases. These time-varying BSL intensity and pressure data were simultaneously measured every 0.1 seconds and completed within 20 seconds. The time and shear stress corresponding to maximal BSL were determined as critical time and CSS, respectively^15,21,22^.

RBC deformability was measured with Rheoscan-D (Sewon meditech, Seoul, Korea) and expressed using the elongation index (EI) when RBC was exposed to shear stress of 3 Pascal (EI@3Pa, %) as reported previously^7^. Fibrinogen, EI@3Pa, and fibrinogen/EI@3Pa were considered as haemorheologic parameters to compare the efficacy with CSS.

### Statistical analysis

All statistical analyses were performed using SPSS (version 21.0, IBM Inc., Chicago, IL, USA). The baseline characteristics were presented as mean ± standard deviation (SD) values for continuous variables and as frequencies with percentages for categorical variables. The statistical significance of differences in continuous variables between two groups and among three groups were determined through an independent sample T-test and a one-way ANOVA, respectively. The statistical significance of differences in categorical variables was determined using Pearson’s Chi-square test. Multiple logistic regression analysis was performed to assess the influence of CSS on diabetic micro-vascular complications after adjustment for covariates. A receiver operating characteristic (ROC) curve was performed to analyse the cut-off value, sensitivity, and specificity of CSS in predicting diabetes-related micro-vascular complications. A P-value less than 0.05 was considered statistically significant.

## Results

### Baseline characteristics

The mean age of all participants was 58.14 ± 11.50 years and the mean duration of diabetes was 8.27 ± 7.98 years. The percentage of males was 59.5%. The mean BMI was 24.76 ± 6.85 kg/m^2^ and the mean HbA1c was 8.27 ± 2.14%. The prevalence of micro-vascular complications was 34.8% for DR, 18.1% for DKD, and 17.9% for DPN.

**Figure 1:**
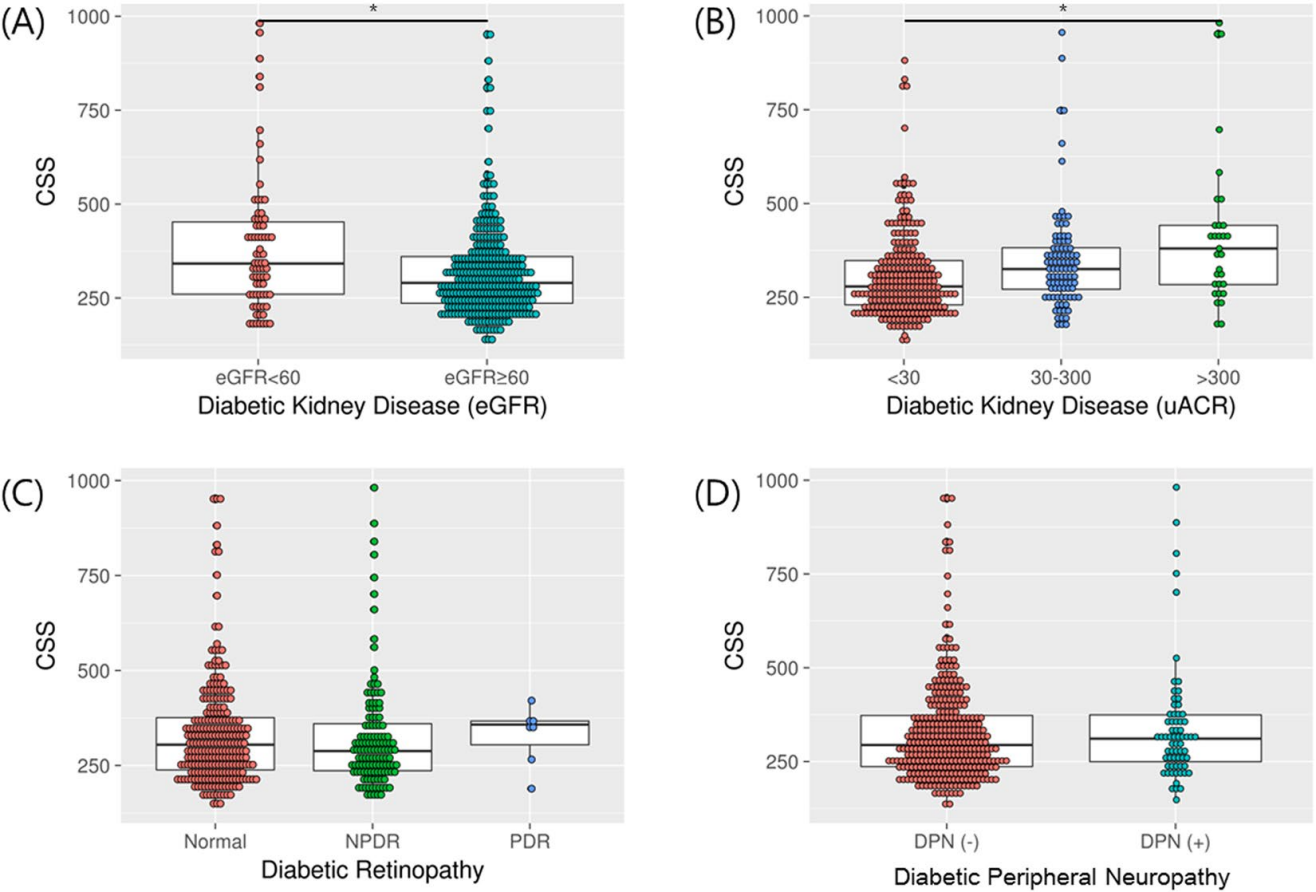
Comparison of CSS values according to diabetic microvascular complications. NPDR, non proliferative diabetic retinopathy; PDR, proliferative diabetic retinopathy; DPN, diabetic polyneuropathy *p < 0.001.

A comparison of CSS values, according to diabetic micro-vascular complications, is presented in Fig. 1. There was a significant relationship between CSS and DKD but not DR or DPN. CSS differed significantly among groups classified as either eGFR (≥60, <60 mL/min/1.73 m^2^) or uACR (<30, 30–300, >300 mg/g) (p < 0.001, both). Within Pearson’s correlation analysis, CSS showed a significant negative correlation with eGFR (r^2^ = 0.023, p = 0.003) and a positive correlation with uACR (r^2^ = 0.138, p < 0.001) (Supplementary 1).

Baseline characteristics of patients, based on eGFR, are presented in Table 1. Participants were divided into two groups; eGFR <60 mL/min/1.73 m^2^ (n = 345) and eGFR ≥60 mL/min/1.73 m^2^ (n = 76). The eGFR <60 group was significantly older (p < 0.001), had a longer duration of diabetes (p < 0.001), a higher proportion of hypertension (p < 0.001), a lower value of Hb and HDL-Cho (p < 0.001 and p = 0.020), and a higher value of ESR and fibrinogen (p < 0.001 and p = 0.018). CSS was significantly higher in the eGFR <60 group compared with the eGFR ≥60 group (385.22 ± 182.89 vs. 317.43 ± 125.11 mPa, p < 0.001). Whereas EI@3Pa was not significantly different between the two groups (30.60 ± 1.91 vs. 30.42 ± 20.54%, p = 0.481), fibrinogen/EI@3Pa was marginally higher in eGFR <60 group (1038.28 ± 197.76 vs. 1141.44 ± 268.71 mg/dL%, p = 0.045). The proportion of those with DR, DPN and coronary artery disease (CAD) was higher in the eGFR <60 group (p = 0.019, 0.004 and <0.001).

**Table 1:** Baseline Characteristics of Patients based on eGFR (n = 421).

	eGFR (mL/min/1.73 m^2^)		P-value
	≥60 (n = 345)	<60 (n = 76)	
Sex (M:F)	1.5:1	1.30:1	0.588
Age (Yrs)	56.36 ± 11.11	66.03 ± 9.85	<0.001
BMI (Kg/m^2^)	24.99 ± 7.47	23.84 ± 3.43	0.070
Diabetes duration (Yrs)	7.16 ± 7.22	13.69 ± 9.37	<0.001
HTN (n(%))	180 (53.4)	62 (82.7)	<0.001
FPG (mg/dL)	165.64 ± 52.34	176.35 ± 67.27	0.299
HbA1c (%)	8.19 ± 2.02	8.77 ± 2.67	0.098
HOMA-IR	4.52 ± 3.79	4.51 ± 2.50	0.981
HOMA-B	48.19 ± 37.72	46.89 ± 46.92	0.860
Hb (g/dL)	14.37 ± 1.54	12.51 ± 1.86	<0.001
T-Cho (mg/dL)	181.27 ± 44.23	173.26 ± 55.29	0.241
HDL-Cho (mg/dL)	51.76 ± 13.91	47.10 ± 15.54	0.020
LDL-Cho (mg/dL)	96.12 ± 38.66	90.04 ± 46.38	0.299
TG (mg/dL)	169.61 ± 110.06	184.16 ± 114.05	0.322
ESR (mm/H)	20.01 ± 20.83	40.67 ± 28.80	<0.001
hsCRP (mg/dL)	0.28 ± 0.71	0.57 ± 1.30	0.099
Fibrinogen (mg/dL)	311.21 ± 54.78	338.44 ± 58.57	0.018
CSS (mPa)	317.43 ± 125.11	385.22 ± 182.89	<0.001
EI@3Pa (%)	30.60 ± 1.91	30.42 ± 20.54	0.481
Fibrinogen/EI@3Pa (mg/dL%)	1038.28 ± 197.76	1141.44 ± 268.71	0.045
DR (%)	32.0	46.5	0.019
DPN (%)	15.4	29.3	0.004
CAD (%)	8.4	23.7	<0.001

BMI, body mass index; HTN, hypertension; FPG, fasting plasma glucose; HbA1c, glycated hemoglobin; HOMA-IR/B, homeostasis model assessment of insulin resistance/beta-cell function; Hb, hemoglobin; T-cho, total-cholesterol; HDL/LDL-cho, high/low density lipoprotein cholesterol; TG, triglyceride; ESR, erythrocyte sedimentation rate; hs-CRP, high sensitivity c-reactive protein; CSS, critical shear stress; EI@3Pa, elongation index at 3pascal; DR, diabetic retinopathy; DPN, diabetic peripheral neuropathy; CAD, coronary artery disease.

Baseline characteristics of patients, based on uACR, are presented in Table 2. Participants were divided into three groups: uACR <30 mg/g (n = 259), uACR 30–300 mg/g (n = 100), and uACR >300 mg/g (n = 31). Compared with the uACR <30 group, uACR ≥30 groups had a significantly longer duration of diabetes (p for trend <0.001), higher systolic and diastolic blood pressure (p for trend <0.001 and 0.001), a higher value of fasting plasma glucose, HbA1c, T-cho, LDL-Cho, ESR, hs-CRP, and fibrinogen (p for trend = 0.012, <0.001, 0.001, 0.010, 0.017, 0.027 and 0.001) and a lower value of Hb (p for trend = 0.006). Between uACR ≥30 groups, the uACR >300 group had a significantly longer diabetes duration and a higher value of T-cho, LDL-cho, fibrinogen, CSS and Fbd/EI@3Pa. CSS showed a gradual increase with deterioration of uACR (308.34 ± 118.14 vs. 349.13 ± 137.35 vs. 422.45 ± 212.72 mPa, p for trend <0.001). With the deterioration of uACR, EI@3Pa decreased (30.63 ± 18.75 vs. 30.57 ± 19.91 vs. 29.64 ± 27.27%, p for trend = 0.027) and fibrinogen/EI@3Pa increased (1022.31 ± 192.56 vs. 1064.36 ± 182.01 vs. 1289.07 ± 319.01 mg/dL%, p for trend <0.001). The proportion of those with DR also increased as uACR worsened (p for trend <0.001).

**Table 2:** Baseline characteristics of patients based on uACR(n = 390).

	uACR (mg/g)			P for trend
	<30 (n = 259)	30–300 (n = 100)	>300 (n = 31)	
Sex (M:F)	1.23:1	1.56:1	2.88:1	0.104
Age (Yrs)	57.78 ± 11.20	58.98 ± 11.84	57.32 ± 11.69	0.625
BMI (Kg/m^2^)	24.62 ± 3.38	25.18 ± 12.32	25.12 ± 5.44	0.813
Diabetes duration (Yrs)	7.35 ± 7.20	8.82 ± 8.95*	14.12 ± 9.12^¶^	<0.001
SBP (mmHg)	129.15 ± 15.37	136.94 ± 14.56*	137.20 ± 21.189*	<0.001
DBP (mmHg)	76.58 ± 10.65	80.65 ± 10.76*	81.63 ± 12.20*	0.001
FPG (mg/dL)	163.56 ± 52.73	172.47 ± 51.38	200.50 ± 81.63*	0.012
HbA1c (%)	7.98 ± 1.93	8.914 ± 2.28	8.88 ± 2.68*	<0.001
HOMA-IR	4.45 ± 3.37	4.96 ± 4.65	4.20 ± 1.89	0.957
HOMA-B	47.61 ± 34.96	51.73 ± 51.40	31.95 ± 21.56	0.204
Hb (g/dL)	14.24 ± 1.59	13.83 ± 1.84*	13.31 ± 2.28	0.006
T-Cho (mg/dL)	179.90 ± 41.92	169.71 ± 48.04*	204.81 ± 65.20^¶^	0.001
HDL-Cho (mg/dL)	52.67 ± 14.01	48.70 ± 13.93	53.15 ± 14.32*	0.051
LDL-Cho (mg/dL)	95.25 ± 35.84	86.10 ± 43.92	110.43 ± 55.21^¶^	0.010
TG (mg/dL)	163.77 ± 104.92	174.36 ± 127.64	206.16 ± 113.86	0.126
ESR (mm/H)	22.71 ± 23.31	27.45 ± 27.85	51.00 ± 22.91*	0.017
hs-CRP (mg/dL)	0.22 ± 0.45	0.49 ± 1.08*	0.44 ± 1.53	0.027
Fibrinogen (mg/dL)	308.55 ± 55.16	318.47 ± 48.96*	363.60 ± 61.64^¶^	0.001
CSS (mPa)	308.34 ± 118.14	349.13 ± 137.35*	422.45 ± 212.72*^¶^	<0.001
EI@3Pa (%)	30.63 ± 18.75	30.57 ± 19.91*	29.64 ± 27.27	0.027
Fibrinogen/EI@3Pa (mg/dL%)	1022.31 ± 192.56	1064.36 ± 182.01*	1289.07 ± 319.01^¶^	<0.001
DR (%)	27.6	46.3	54.8	<0.001
DPN (%)	14.7	23.5	22.6	0.111
CAD (%)	10.4	11	16.1	0.632

BMI, body mass index; SBP, systolic blood pressure; DBP, diastolic blood pressure; FPG, fasting plasma glucose; HbA1c, glycated hemoglobin; HOMA-IR/B, homeostasis model assessment of insulin resistance/beta-cell function; Hb, hemoglobin; T-cho, total-cholesterol; HDL/LDL-cho, high/low density lipoprotein cholesterol; TG, triglyceride; ESR, erythrocyte sedimentation rate; hs-CRP, high sensitivity c-reactive protein; CSS, critical shear stress; EI@3Pa, elongation index at 3pascal; DR, diabetic retinopathy; DPN, diabetic peripheral neuropathy; CAD, coronary artery disease.

*p < 0.05 vs. uACR <30 group, ^¶^p < 0.05 vs. uACR 30–300 group by post-hoc analysis.

### CSS and risk of DKD

The odds ratio(OR) of CSS for DKD was analysed using multiple logistic regression analysis (Table 3). CSS was analysed as continuous variables and terciles. The mean CSS values from the lowest (T1) to the highest tertile (T3) were 216.67 ± 28.20, 298.81 ± 25.14 and 475.44 ± 148.61, respectively. Within terciles, T1 was set as the reference range. As dependent variables, eGFR <60 ml/min/1.73 m^2^ and uACR ≥30 mg/g were used. Logistic regression models were adjusted for, as following: model 1, non-modifiable risk factors (age, sex); model 2, and model 1, plus risk factors related to diabetes complications (diabetes duration, hypertension); and model 3, model 2, plus confounding serologic abnormalities (Hb).

**Table 3:** Odds ratios(95% CI) for DKD according to CSS.

Dependent variable: eGFR <60 ml/min/1.73 m^2^				
	crude	Model1	Model2	Model3
CSS	1.003(1.001–1.005) *	1.003(1.002–1.005)*	1.004(1.002–1.006)*	1.003(1.001–1.005)*
CSS T1	1(ref)	1(ref)	1(ref)	1(ref)
CSS T2	1.101(0.554–2.191)	1.398(0.673–2.902)	1.202(0.537–2.691)	2.010(0.793–5.089)
CSS T3	2.265(1.207–4.253)*	2.727(1.383–5.377)*	2.134(0.983–4.632)	2.573(1.057–6.264)*
**Dependent variable: uACR ≥ 30 mg/g**				
	**crude**	**Model1**	**Model2**	**Model3**
CSS	1.003(1.001–1.005)*	1.003(1.002–1.005)*	1.003(1.002–1.005)*	1.003(1.001–1.005)*
CSS T1	1(ref)	1(ref)	1(ref)	1(ref)
CSS T2	1.638(0.927–2.896)	1.641(0.925–2.912)	1.344(0.723–2.499)	1.451(0.772–2.730)
CSS T3	3.046(1.743–5.324)*	3.244(1.841–5.716)*	3.129(1.684–5.815)*	3.063(1.632–5.748)*

Model 1, adjusted for Age, Gender; Model 2, adjusted as in Model 1 plus diabetes duration and hypertension; Model 3, adjusted as in Model 2 plus Hb.

*p < 0.05, T, tercile; Ref, reference range.

The OR of CSS as a continuous variable for DKD was 1.003 in model 3 (95% CI = 1.001–1.005, p < 0.05 dependent on eGFR, and 95% CI = 1.001–1.005, p < 0.01 dependent on uACR). When CSS was divided into tertiles, T3 was independently associated with the risk of DKD in model 3 compared with T1 (OR = 2.573, 95% CI = 1.057–6.264, p < 0.05 dependent on eGFR and OR = 3.063, 95% = CI 1.632–5.748, p < 0.01 dependent on uACR). Therefore, the risk of DKD rose 2.5–3.0 times if the CSS level was higher in the highest tertile compared to the lowest tertile.

In the ROC curve analysis, the cut-off value of CSS was 312.67 mPa dependent on eGFR (area under curve(AUC) = 0.615, sensitivity 60.3%, specificity 59.6%) and 309.06 mPa dependent on uACR (AUC = 0.635, sensitivity 60.2%, specificity 60.3%) (Supplementary 2).

## Discussion

In our present study, the patients with higher CSS levels were more likely to have DKD. In addition to CSS, the common risk factors for DKD in both eGFR and uACR standards were the duration of diabetes, presence of hypertension, Hb value, ESR, fibrinogen, and the presence of DR. After adjusting for age, sex, duration of diabetes, presence of hypertension, and Hb, the risk of developing DKD was approximately 2.5–3.0 times greater in the highest CCS tertile than in the lowest, with statistical significance.

Currently, the uACR measured in a fresh, first morning, spot sample is preferred as a screening tool for DKD. Compared to urinary total protein, urine albumin measurement provides a more specific and sensitive measure of changes in glomerular permeability^17^. However, there is a disadvantage in that uACR is recommended for repeated testing because the results vary according to the patient’s exercise, upright posture, and condition of infection or the sample’s storage temperature^17,23^. Additionally, approximately 20% to 63% of patients with low eGFR(<60 mL/min/1.73 m^2^) were reported to be normoalbuminuric^24^. For this reason, new potential novel biomarkers for early detection of DKD have been suggested, targeting several pathogeneses of DKD, including hyperfiltration, inflammation, and renal remodelling^25^. Recently, oxidative stress has emerged as a new pathophysiology of DKD which eventually alters haemodynamics^26,27^. Among the alterations in haemodynamics, reduced RBC deformability and increased RBC aggregation have been strongly featured and implicated in the pathogenesis of diabetic micro- and macro-vascular complications^28,29^.

There have been several studies reporting the relationship between haemorheologic markers and diabetic micro-vascular complications, and the comparison with the present study is as follows. First, DR was associated with impairment of RBC deformability^7^, plasma fibrinogen, or conventional aggregation indices^30^; however, no significant differences in haemorheologic markers between NPDR and PDR were noted^30^. In our study, there was no significant difference in CSS among normal, NPDR, and PDR numbers. This appears to be due to the small number of patients diagnosed with PDR in our study. In addition, since impairment of RBC deformability precedes RBC aggregation^5^ and DR precedes DKD^17^, the association of RBC deformability might be strong in DR, whereas the association of CSS is strong in DKD. Second, moderately increased albuminuria (uACR 30–300 mg/g) was significantly associated with impairment of RBC deformability^9^ or fibrinogen divided by RBC deformability^8^, compared with uACR <30 mg/g, but CSS showed only a significant difference between severely increased albuminuria (uACR >300 mg/g) and uACR <30 mg/g^8^. Our study provides additional evidence of CSS as a significant differential marker for moderately increased albuminuria (uACR 30–300 mg/g). Besides, CSS was the most significant indicator for DKD among the haemorheologic indices; we confirmed again that CSS is an independent hemorheologic index reflecting the synergistic effect of reduced RBC deformability and increased fibrinogen^31^. Third, the increased impairment of RBC deformability was noted in DPN without statistical significance^7^. In our study, CSS also increased without statistical significance.

CSS is one of several indices that represents RBC aggregation. CSS has an advantage in that it does not require haematocrit adjustments, unlike the conventional aggregation indices^15^, and fibrinogen does not affect the value of CSS if it is measured from the BSL of a transient microfluidic aggregometer^22^. Additionally, it has a similar trend to changes in whole blood viscosity with temperature variations^32^. Moreover, the new role of RBCs in coagulation has been recognised. A recent study reported that increments in CSS significantly increased platelet activation while RBC deformability was not associated^33^. The enhanced aggregation and the induced central compaction of RBC favours the migration of platelets to the marginal flow zone and modulates the possibility of platelet activation^16^, which causes vascular occlusion. Therefore, CSS may be used as a novel biomarker of hemorheological risk in both diabetic microcirculation and seasonal ischemic macro-vascular diseases.

The cut-off value of CSS for detecting DKD was approximately 310 mPa in our study (data was not shown). In previous studies, the mean CSS in the channel flow has been reported as 200.5 mPa^15^. Interestingly, an approximately 30% increase in CSS in acute coronary syndrome was noted: 265 mPa in stable angina, 338 mPa in unstable angina, and 324 mPa in acute myocardial infarction^34^. Further studies to establish the relationship between increased CSS values and diabetic vascular complications, and the study of each cut-off value as a screening tool, may be worthwhile.

To the best of our knowledge, this is the first study that has revealed the relationship between CSS and early stage DKD and presented the CSS cut-off values for DKD. However, there were some limitations to our study. This study was designed retrospectively and was a cross-sectional study; therefore, a causal relationship was hard to determine. In addition, omitted variable bias might have occurred due to the lack of important confounding variables in the regression analysis, such as medication. Further research with consideration to usage of cardiovascular medication is needed. With regard to hemorheologic parameters, we used the measured value once and did not use the average value from the repeated measurement. Although the mean value may more accurately reflect the hemorheologic change, single measurement could be acceptable based on previous studies^7–10^. Well-designed, prospective studies with a larger sample size are warranted in the future.

In conclusion, the elevation of CSS was closely associated with an increased risk of DKD. These results reinforce the possibility that RBC aggregability might contribute to DKD development. We anticipate that if additional studies and reference ranges are accumulated, haemorheologic parameters, including CSS, may have a role as screening tools for diabetic micro-vascular complications.

## Electronic supplementary material

Supplementary Information
