# Author Correction: Critical Shear Stress is Associated with Diabetic Kidney Disease in Patients with Type 2 Diabetes

**DOI:** 10.1038/s41598-018-25252-8

**Published:** 2018-04-30

**Authors:** Seung Min Chung, Jung Hyun Oh, Jun Sung Moon, Yu Kyung Kim, Ji Sung Yoon, Kyu Chang Won, Hyoung Woo Lee

**Affiliations:** 1 Division of Endocrinology and Metabolism, Department of Internal Medicine, Yeungnam College of Medicine, Daegu, Republic of Korea; 2 Kwon and Oh Internal Medicine, Sangju, Gyeongbuk, Republic of Korea; 3 Department of Clinical Pathology, School of Medicine, Kyungpook National University, Daegu, Republic of Korea

Correction to: *Scientific Reports* 10.1038/s41598-018-19274-5, published online 17 January 2018

In the original version of this Article, Affiliation 3 was incorrectly listed as ‘Department of Clinical Pathology, Kyungpook National University School of Medicine, Daegu, Republic of Korea.’ The correct affiliation is listed below:

Department of Clinical Pathology, School of Medicine, Kyungpook National University, Daegu, Republic of Korea.

This error has now been corrected in the PDF and HTML versions of the Article.

