# Supplementary material for: Critical Shear Stress is Associated with Diabetic Kidney Disease in Patients with Type 2 Diabetes
Source: Sci Rep. 2018 Jan 17;8:908. doi: 10.1038/s41598-018-19274-5 (PMC5772353; doi:10.1038/s41598-018-19274-5)
Supplement: Supplementary file 1 — Supplementary Information [file 41598_2018_19274_MOESM1_ESM.pdf]

Critical Shear Stress is Associated with Diabetic Kidney Disease in Patients with Type 2 Diabetes

Seung Min Chung, Jung Hyun Oh, Jun Sung Moon, Yu Kyung Kim, Ji Sung Yoon, Kyu Chang Won, Hyoung Woo Lee

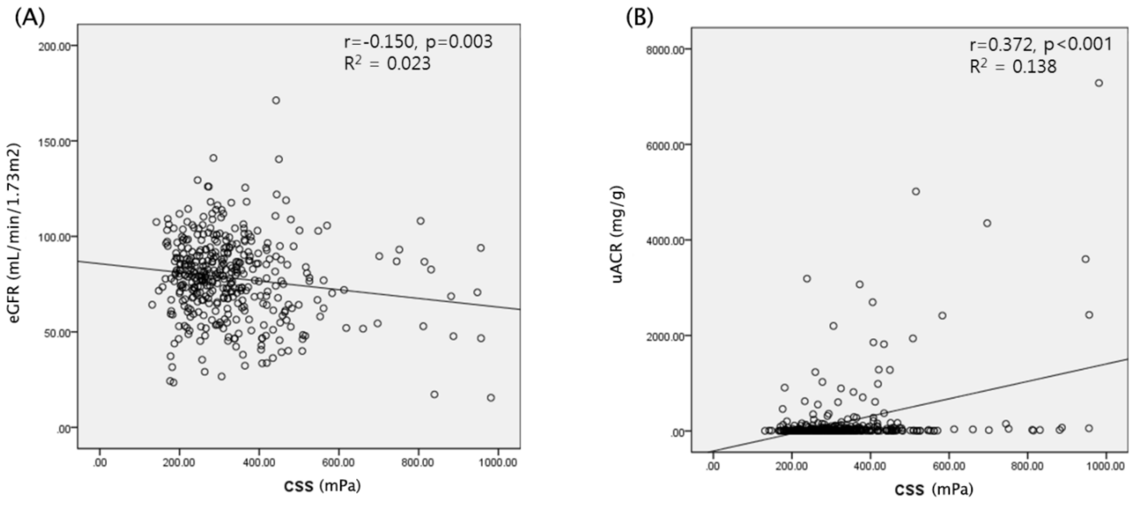

Supplementary 1. Within Pearson's correlation analysis, CSS showed (A) a significant negative correlation with eGFR (r2=0.023, p=0.003) and (B) a positive correlation with uACR (r2=0.138, p<0.001).

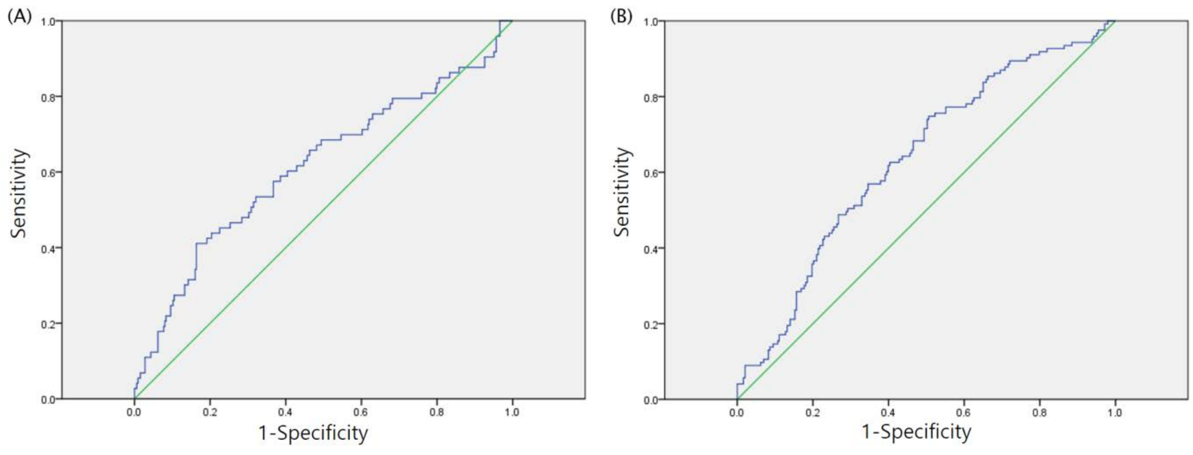

Supplementary 2. In the ROC curve analysis, the cut-off value of CSS was (A) 312.67 mPa dependent on eGFR (area under curve(AUC)=0.615, sensitivity 60.3%, specificity 59.6%) and (B) 309.06 mPa dependent on uACR (AUC=0.635, sensitivity 60.2%, specificity 60.3%).
